# Micronutrient status and fatty acid profile of adults with SARS-CoV-2 infection—an observational study

**DOI:** 10.3389/fnut.2025.1608300

**Published:** 2025-09-16

**Authors:** Melanie Bleffgen, Laura Schinhammer, Francesco Giuseppe De Rosa, Silvia Scabini, Beate Brandl, Hans Hauner, Simona Bo, Thomas Skurk

**Affiliations:** ^1^Core Facility Human Studies, ZIEL—Institute for Food and Health, Technical University of Munich,, Freising, Germany; ^2^Department of Medical Sciences, University of Torino, Torino, Italy; ^3^Else Kröner-Fresenius-Center of Nutritional Medicine, Technical University of Munich,, Munich, Germany; ^4^School of Medicine and Health, Institute of Nutritional Medicine, Technical University of Munich,, Munich, Germany

**Keywords:** dried blood spots, micronutrient status, vitamin D, fatty acid profile, SARS-CoV-2, COVID-19, observational study, polyunsaturated fatty acids (PUFAs)

## Abstract

**Introduction:**

SARS-CoV-2 infection is a complex disease with multiple dimensions, involving factors that promote infection and virus-driven processes in many body organs. The micronutrient status, beyond others, acts as a potential confounder, influencing susceptibility to infection and disease severity. Additionally, the virus appears to alter lipid metabolism, which may serve a dual function, suppor viral replication while simultaneously contributing to the body’s defense and repair mechanisms.

**Methods:**

This observational study compared micronutrient levels (vitamin D, selenium, zinc, magnesium, and iron) and lipid profiles between 139 SARS-CoV-2 -positive patients (62 hospitalized, 77 home care) and 314 healthy controls, using dried blood spots. We also examined differences by treatment setting (hospitalized vs. home care) as a proxy for disease severity.

**Results:**

Patients with SARS-CoV-2 infection exhibited similar micronutrient levels but showed a significantly impaired lipid profile compared to healthy controls. Notably, there was a significant decrease in palmitic (p-value < 0.01) and stearic acid levels (*p*-value < 0.01) and a significant increase in omega-3 and omega-6 PUFAs, like AA (*p*-value < 0.01), DHA (*p*-value < 0.01), and EPA (*p*-value < 0.05) were detected. In the SARS-CoV-2 positive cohort, hospitalized patients had significantly lower micronutrient levels (*p* < 0.01 for all measured micronutrients) compared to those receiving home care.

**Discussion:**

These findings suggest that SARS-CoV-2 infection alters lipid metabolism and that lower micronutrient status may be linked to greater disease severity.

## 1 Introduction

The COVID-19 pandemic has been a global challenge. While clinical outcomes vary widely, emerging evidence highlights the role of nutritional status, particularly micronutrients and lipids, in modulating susceptibility to infection and disease progression (1–3).

Micronutrients such as vitamin D, selenium, zinc, magnesium, and iron are critical to maintaining a balanced immune response (4). They influence antiviral defense through mechanisms including cytokine regulation, oxidative stress reduction, and enhancement of T- and B-cell activity (3, 5, 6).

Many studies show that an adequate micronutrient status supports overall health, while deficiencies are linked to an elevated infection risk and more severe disease progression (7–10), especially with COVID-19 (11–13).

In parallel, fatty acids, especially polyunsaturated fatty acids (PUFAs), not only constitute structural components of cell membranes but also act as mediators of inflammation and immune function (14, 15). In viral infections, including COVID-19, disruptions in lipid metabolism have been observed, as the virus exploits host lipid resources to support replication (14, 16, 17). Some studies have shown that SARS-CoV-2 induces virus mediated changes in the metabolome and lipidome, which are crucial for viral replication and the immune response (14, 18–20). For example, studies on COVID-19 patients consistently reported increased PUFAs and decreased saturated fatty acids levels (14, 18, 19).

In this study, wie aimed to determine whether SARS-CoV-2 infection is associated with alterations in both micronutrient status and fatty acid composition. To this end, we compared adults with confirmed SARS-CoV-2 infection, stratified by hospitalization status, with healthy controls. We hypothesized that infection would be accompanied by shifts in micronutrient and lipid profiles, with more pronounced changes in hospitalized patients.

Identifying such changes may enhance understanding of COVID-19 progression and inform targeted micronutrient screening and preventive strategies.

## 2 Methods

Procedures for the observation study were followed in accordance with the ethical standards of the Helsinki Declaration and were approved by the ethics committee of the School of Medicine and Health of the Technical University of Munich, Germany (655/20 S). All study participants provided online approval. The study was registered in the German Clinical Trials Register (DRKS): DRKS00022514.

### 2.1 MeDiCo Health study cohort

Participant enrollment for this cross-sectional observation study started on November 23^rd^, 2020 and continued until December 14^th^, 2021. Therefore, volunteers either responded to flyers posted in doctors’ offices and hospitals in Germany or were recruited from the “Città della Salute e della Scienza” hospital in Italy.

**Figure 1:**
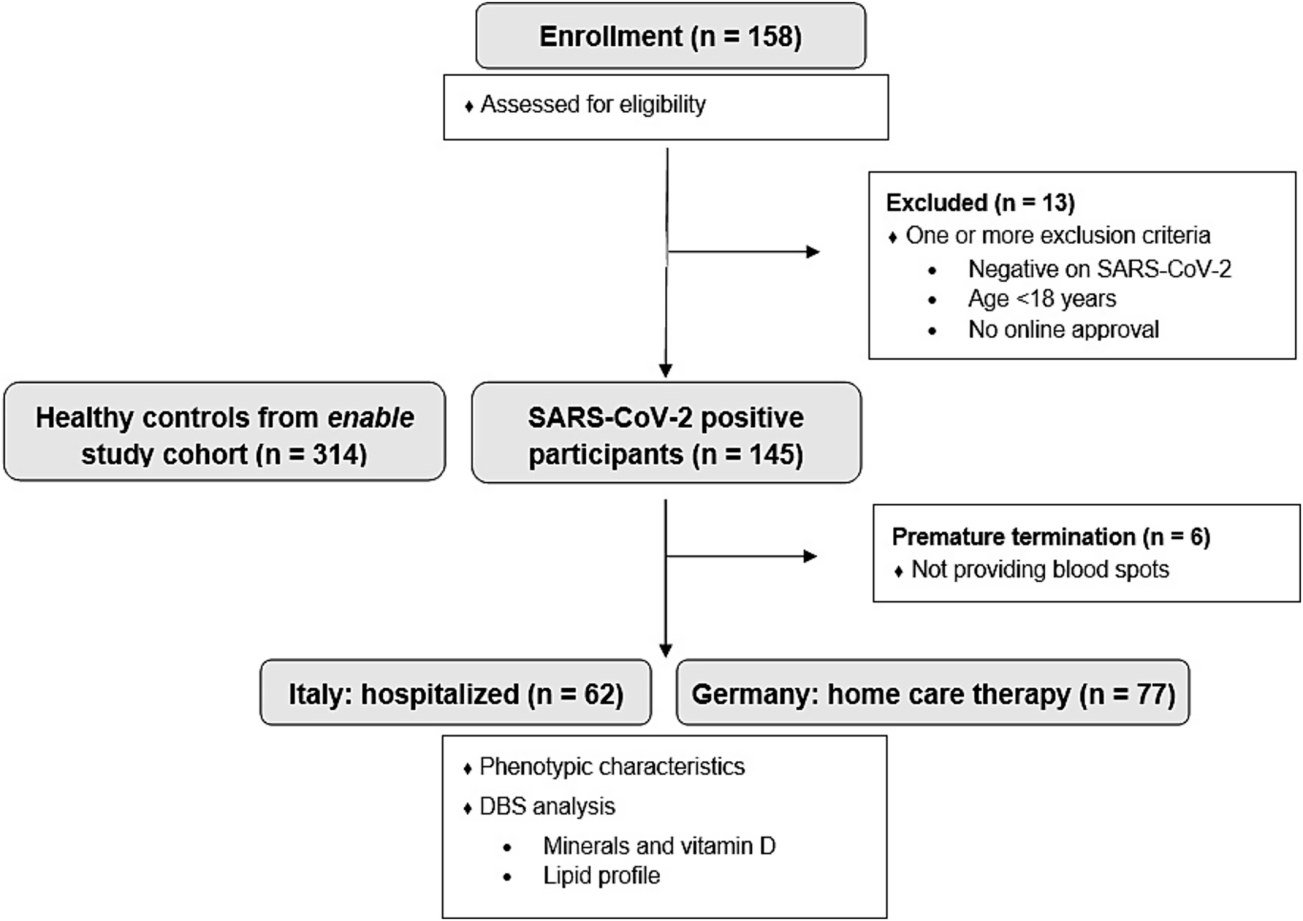
Participant flow diagram of the MeDiCo Health study, illustrating the inclusion of SARS-CoV-2 positive participants from Germany (receiving home care therapy) and Italy (hospitalized). Additionally, the diagram includes the healthy control group from the enable study cohort.

The participants’ eligibility was assessed with a screening questionnaire. A total of 158 volunteers were initially screened. Exclusion criteria included age under 18 years, a negative SARS-CoV-2 test, or a positive test older than 7 days. SARS-CoV-2 infection was confirmed by antigen testing. All participants underwent nasopharyngeal swab testing, followed by PCR testing in the clinical setting or rapid antigen testing when performed at home. Participants who did not complete the study or failed to provide dried blood spot (DBS) samples were also excluded. The final study population consisted of 139 participants (62 hospitalized in Italy, 77 home care in Germany). A detailed participant flow chart is presented in Figure 1. None of the participants had been vaccinated against SARS-CoV-2 during the study period.

### 2.2 Study design

This was an observational cross-sectional study among adults with an acute SARS-CoV-2 infection located in Germany and Italy.

### 2.3 Dried blood spot analysis in whole blood

The decision to use DBS was driven by the exceptional circumstances of the COVID-19 pandemic, where contact-free, low-burden sampling was essential for participant safety and feasibility of multi-site recruitment. DBS is a well-established approach in large epidemiological and nutritional studies, and its applicability for the quantification of fatty acids and several micronutrients has been demonstrated in prior validation studies (21–23).

After inclusion into the study, a home test kit with a detailed explanation for collecting DBS was send from the Core Facility Human Studies of the ZIEL- Institute for Food & Health, Freising, Germany to the participants. Participants collected DBS (filter paper: Whatman™, Cytiva, Maidstone, UK) from their fingertips at the beginning of the study with a home test kit. DBS were subsequently sent to Vitas AS (Oslo, Norway) for the analysis of selenium [μg/mL], zinc [μg/mL], vitamin D [nmol/L], iron [μg/mL], magnesium [μg/mL] and lipid profile expressed in % of fatty acid methyl esters (FAME). The lipid profile includes palmitic acid (C16:0), stearic acid (C18:0), oleic acid (C18:1c9), linoleic acid (LA, C18:2n6), linolenic acid (ALA, C18:3n3), *γ*-linolenic acid (GLA, C18:3n6), dihomogammalinolenic acid (DGLA, C20:3n6), arachidonic acid (AA, C20:4n6), eicosapentaenoic acid (EPA, C20:5n6), docosapentaenoic acid (DPA, C22:5n3), docosahexaenoic acid (DHA, C22:6n3), omega-6 to −3 fatty acid quotient. The analyzing laboratory Vitas AS defined the normal range as the 95% reference interval, corresponding to the 2.5th and 97.5th percentiles. Participants were instructed to fast overnight before sample collection for accurate lipid profiling.

### 2.4 Healthy controls (*enable* study cohort)

We also took advantage of data from the *enable* cohort (24) to compare the SARS-CoV-2 positive MeDiCo Health cohort with healthy participants (SARS-CoV-2 negative). The *enable* study participants underwent a phenotyping program for the different age cohorts included, beyond others, anthropometry, body composition analysis, health and functional status, and assessment of dietary intake including food preferences and aversions. 459 healthy volunteers from different age groups including young adults (18–25 years; *n* = 94), middle-aged adults (40–65 years “middle-agers,” *n* = 205), and older adults (75–85 years; *n* = 160) underwent this program. Further details on the study design and the characteristics of the cohorts can be found elsewhere (24). For the current study, we used data from “middle-agers” and older adults (*n* = 314) as healthy controls, as these cohorts had a similar age range compared to the MeDiCo Health participants (see Figure 1). DBS data with the same blood parameters were used for comparison.

### 2.5 Data analysis and statistics

Data were analyzed in Excel 2016 and GraphPad Prism 10.4.0. Results are presented as mean ± SD. Box plots in Tukey’s style were used for the graphical presentation. *p*-values < 0.05 were regarded as statistically significant. Shapiro–Wilk test and quantil-quantil-plots were used to test normal distribution of data. For comparisons between groups, either a paired *t*-test or the Mann–Whitney test was applied to assess statistical significance.

Given the exploratory and cross-sectional nature of our study, we applied a basic statistical approach, focusing on descriptive comparisons and group-level associations rather than complex modeling. As some analytes were either not measurable or fell below the detection limit in the DBS analysis, the exact number of samples analyzed is indicated in the respective table or figure.

## 3 Results

### 3.1 MeDiCo Health study cohort: baseline and phenotypical information

The MeDiCo Health study cohort included a total of 139 SARS-CoV-2-positive participants, comprising 77 individuals from Germany and 62 from Italy. All German participants underwent home care therapy, while all Italian participants were hospitalized. The need for inpatient treatment in the Italian cohort indicates a more severe course of SARS-CoV-2 infection. Among the hospitalized participants, some had comorbidities such as cardiovascular disease, cancer, or obesity and six passed away during the study period. Table 1 outlines the baseline and phenotypical characteristics of the MeDiCo Health study cohort.

**Table 1:** Baseline and phenotypical information from MeDiCo Health study cohort.

	Number of participants, n	% from all participants
Baseline information		
SARS-CoV-2 positive	139	100
Study site		
Germany	77	55
Italy	62	45
Therapy		
Home care	77	55
Hospitalized	62	45
Deceased		
Home care	0	0
From hospitalized	6	4
Phenotypic information		
Sex		
Female	74	53
Male	65	47
Age, years	51.4 ± 18.9	
BMI, kg/m^2^	25.2 ± 4.4	

Results are presented as absolute numbers and percentages of all participants, unless otherwise indicated. Age and BMI are shown as mean ± SD.

### 3.2 Comparative analysis of micronutrient and fatty acid status in SARS-CoV-2 positive and negative cohorts

The comparison of the micronutrient status between SARS-CoV-2-positive (MeDiCo Health) and SARS-CoV-2-negative (*enable*) study cohorts is shown in Table 2.

**Table 2:** Micronutrient levels in SARS-CoV-2 positive study cohort (*n* = 139; except Vitamin D: *n* = 127) and micronutrient levels in SARS-CoV-2 negative study cohort (*n* = 314).

Micronutrients	SARS-CoV-2 positive	SARS-CoV-2 negative	*p*-value
	MeDiCo Health cohort	*enable* cohort	
Magnesium [μg/ml]	35.9 ± 9.7	**31.8 ± 3.6**	<0.01
Iron [μg/ml]	513.6 ± 136.3	523.3 ± 83.4	NS
Zinc [μg/ml]	6.5 ± 1.9	6.4 ± 1.1	NS
Selenium [μg/ml]	0.18 ± 0.04	**0.15 ± 0.03**	<0.01
Vitamin D [nmol/l]	104.1 ± 43.0	67.4 ± 34.3	NS

Results are presented as mean ± SD. P-values < 0.05 were regarded as statistically significant. Shapiro–Wilk test and quantil-quantil-plots were used to test normal distribution of data. For comparisons between two groups, either a paired t-test or Mann–Whitney test was applied to assess statistical significance. Significantly lower values are marked in bold.

The blood values are largely consistent between the two study cohorts, with the exception of significant differences observed in magnesium and selenium levels, both of which were higher in the SARS-CoV-2-positive cohort compared to the SARS-CoV-2-negative cohort.

**Figure 2:**
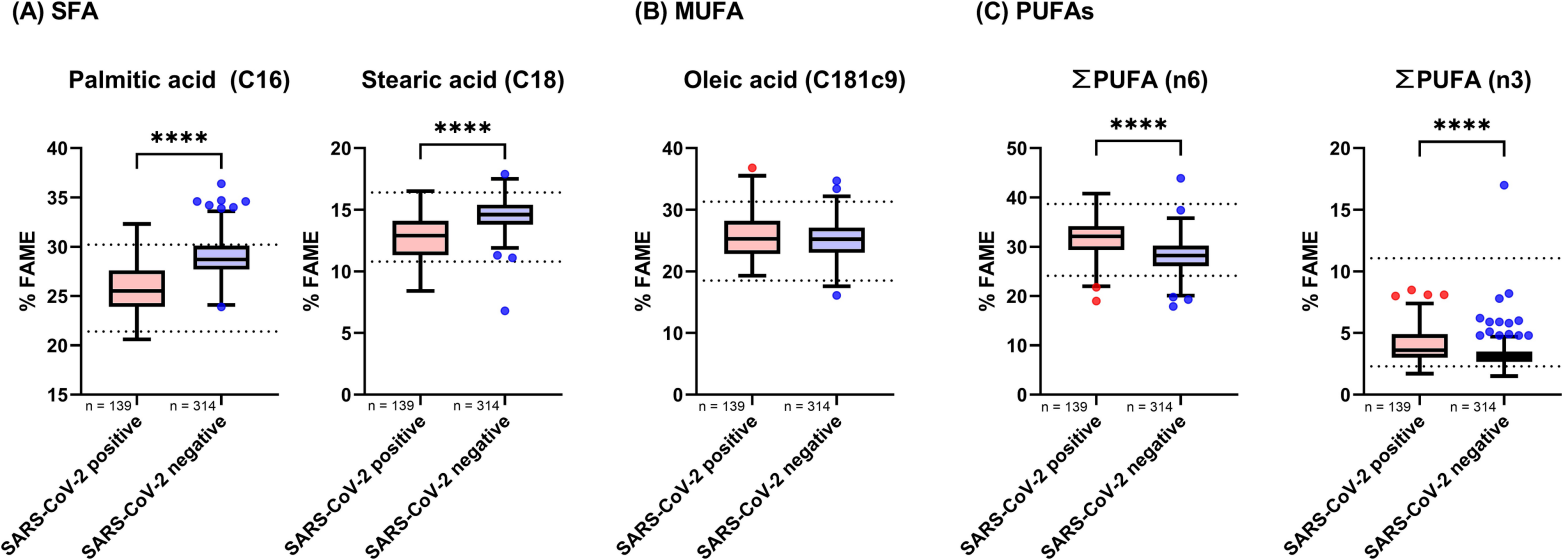
Fatty acid levels in whole blood of SARS-CoV-2 positive and negative study cohorts. Fatty acid concentrations were compared between participants who tested positive and those who tested negative for SARS-CoV-2. Data are presented as Tukey-style boxplots. Statistical significance was determined using a threshold of *p* < 0.05. The Shapiro–Wilk test and quantile-quantile (Q-Q) plots were used to assess the normality of the data distribution. Depending on the distribution and pairing of data, either a paired t-test or the Mann–Whitney U test was employed to evaluate differences between groups. Asterisks denote levels of statistical significance: *p* < 0.05 (), *p* < 0.01 (), *p* < 0.001 (), and *p* < 0.0001 (****). **(A)** Shows levels of saturated fatty acids (SFAs), **(B)** presents monounsaturated fatty acids (MUFAs), and **(C)** displays the combined levels of omega-6 and omega-3 polyunsaturated fatty acids (PUFAs).

The results of the fatty acid analysis are presented in Table 3 and Figure 2. Table 3 provides a detailed overview of all measured fatty acids, while Figure 2 categorizes these fatty acids into their respective subgroups: saturated fatty acids (SFAs), monounsaturated fatty acids (MUFAs), and omega-3 and omega-6 PUFAs. The analysis revealed that SFAs were significantly decreased in the SARS-CoV-2-positive cohort compared to the SARS-CoV-2-negative cohort. In contrast, omega-3 and omega-6 PUFAs were significantly elevated across all measured fatty acids in the SARS-CoV-2-positive cohort.

**Table 3:** Fatty acid levels in SARS-CoV-2 positive study cohort (*n* = 139; except AA: *n* = 127) and fatty acid levels in SARS-CoV-2 negative study cohort (*n* = 314, except AA; EPA, ALA: *n* = 313, GLA: *n* = 290).

Subgroup	Fatty acids (% FAME)	SARS-CoV-2 positive	SARS-CoV-2 negative	*p*-value
		MeDiCo Health cohort	*enable* cohort	
SFA	Palmitic acid (C16)	**25.8 ± 2.4**	29.0 ± 1.9	<0.01
	Stearic acid (C18)	**12.8 ± 1.8**	14.6 ± 1.3	<0.01
MUFA	Oleic acid (C18:1w9)	25.9 ± 3.9	25.2 ± 2.8	NS
PUFA (n6)	LA (C18:2n6)	20.3 ± 3.6	**19.5 ± 2.9**	<0.01
	AA (C20:4n6)	9.5 ± 2.2	**7.1 ± 1.3**	<0.01
	DGLA (C20:3n6)	1.6 ± 0.4	**1.4 ± 0.3**	<0.01
	GLA (C18:3n6)	0.2 ± 0.1	**0.2 ± 0.1**	<0.01
PUFA (n3)	DHA (C22:6n3)	2.3 ± 1.0	**1.7 ± 0.5**	<0.01
	EPA (C20:5n3)	0.5 ± 0.4	**0.5 ± 0.3**	<0.05
	DPA (C22:5n3)	0.9 ± 0.3	**0.8 ± 0.2**	<0.01
	ALA (C18:3n3)	0.3 ± 0.1	**0.3 ± 0.1**	<0.01
∑ PUFA (n6)		31.6 ± 4.0	**28.1 ± 3.3**	<0.01
∑ PUFA (n3)		4.0 ± 1.5	**3.2 ± 1.2**	<0.01
Quotients	O6/O3	**8.8 ± 2.9**	9.3 ± 2.4	<0.05
	AA/EPA	29.1 ± 18.7	**17.0 ± 7.5**	<0.01

Results are presented as mean ± SD. P-values < 0.05 were regarded as statistically significant. Shapiro–Wilk test and quantil-quantil-plots were used to test normal distribution of data. For comparisons between two groups, either a paired t-test or Mann–Whitney test was applied to assess statistical significance. Significantly lower values are marked in bold.

### 3.3 Comparative analysis of micronutrient and fatty acid status according to therapy type

**Figure 3:**
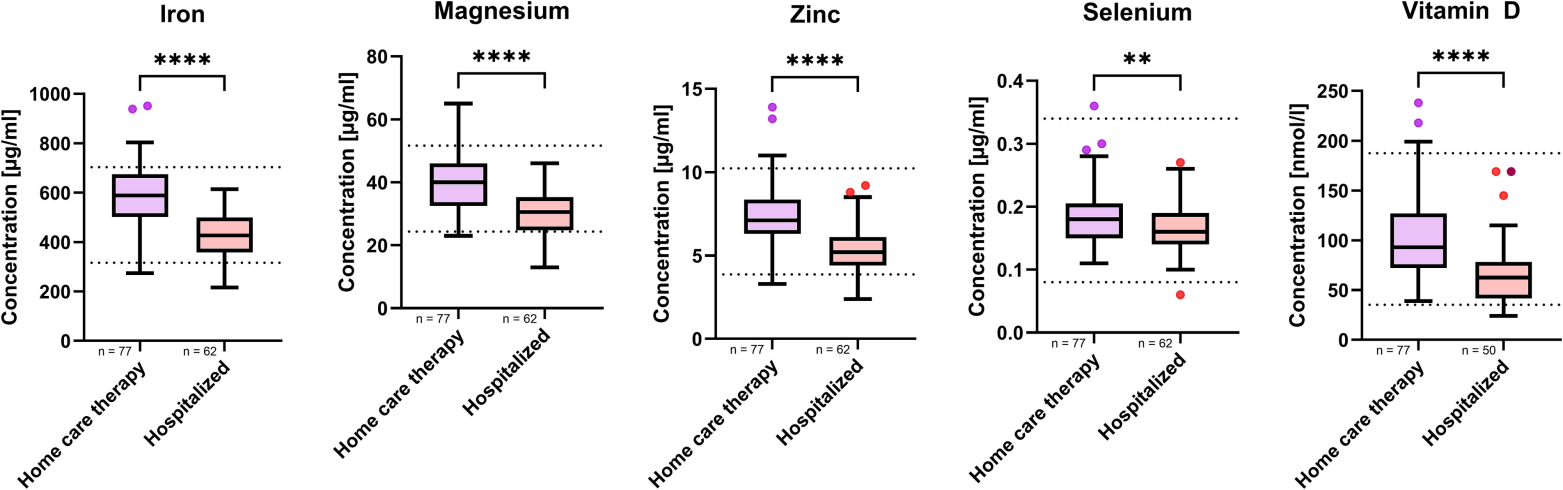
Micronutrient whole blood levels in SARS-CoV-2 positive study cohort stratified by therapy type. n represents the number of participants in respective therapy group. Results are presented as boxplots in style of Tukey. *p*-values < 0.05 were regarded as statistically significant. Shapiro–Wilk test and quantil-quantil-plots were used to test normal distribution of data. For comparisons between two groups, either a paired t-test or Mann–Whitney test was applied to assess statistical significance. The asterisks (*p < 0.05, **p < 0.01, ***p < 0.001, ****p < 0.0001) indicate different levels of significance between study cohorts.

Further analysis, stratifying the SARS-CoV-2-positive MeDiCo Health cohort by therapy type, home care versus hospitalized participants, revealed significantly lower levels of iron, magnesium, zinc, selenium, and vitamin D in hospitalized participants compared to SARS-CoV-2-positive participants receiving home care. The results of this comparison are presented in Table 4 and Figure 3.

**Table 4:** Micronutrient levels in SARS-CoV-2 positive study cohort stratified by therapy type.

Micronutrients	SARS-CoV-2 positive		*p*-value
	Home care therapy	Hospitalized	
Magnesium [μg/ml]	40.0 ± 8.8	**30.7 ± 8.0**	<0.01
Iron [μg/ml]	587.2 ± 120.7	**422.3 ± 92.4**	<0.01
Zinc [μg/ml]	7.3 ± 1.9	**5.3 ± 1.4**	<0.01
Selenium [μg/ml]	0.19 ± 0.04	**0.16 ± 0.04**	<0.01
Vitamin D [nmol/l]	84.7 ± 31.9	**81.9 ± 48.7**	<0.01

Participants with a home care therapy (*n* = 77) compared with hospitalized participants (*n* = 62, except Vitamin D: *n* = 50). Results are presented as mean ± SD. P-values < 0.05 were regarded as statistically significant. Shapiro–Wilk test and quantil-quantil-plots were used to test normal distribution of data. For comparisons between two groups, either a paired t-test or Mann–Whitney test was applied to assess statistical significance. Significantly lower values are marked in bold.

The results of the lipid acid analysis are presented in Table 5. Hospitalized participants exhibited significantly higher levels of palmitic acid and oleic acid compared to those receiving symptomatic home care therapies. Additionally, they showed significantly elevated levels of AA and DHA, as well as a higher AA/EPA ratio. Interestingly, participants undergoing home care therapy had a significantly higher total concentration of omega-6 fatty acids compared to hospitalized participants.

**Table 5:** Micronutrient levels in SARS-CoV-2 positive study cohort stratified by therapy type, either home care (*n* = 77) or hospitalized (*n* = 62, except AA: *n* = 50).

Subgroup	Fatty acids	SARS-CoV-2 positive		
		Home care therapy	Hospitalized	*p*-value
SFA	Palmitic acid (C16)	**25.3 ± 2.5**	26.4 ± 2.1	<0.01
	Stearic acid (C18)	13.7 ± 1.3	**11.6 ± 1.7**	<0.01
MUFA	Oleic acid (C18:1w9)	**23.7 ± 2.8**	28.6 ± 3.3	<0.01
PUFA (n6)	LA (C18:2n6)	22.3 ± 2.8	**17.8 ± 2.8**	<0.01
	AA (C20:4n6)	**9.0 ± 1.9**	10.0 ± 2.4	<0.01
	DGLA (C20:3n6)	1.6 ± 0.3	**1.5 ± 0.4**	<0.01
	GLA (C18:3n6)	0.2 ± 0.1	0.2 ± 0.1	NS
PUFA (n3)	DHA (C22:6n3)	**2.1 ± 1.0**	2.5 ± 1.0	<0.05
	EPA (C20:5n3)	0.6 ± 0.4	**0.4 ± 0.3**	<0.01
	DPA (C22:5n3)	1.0 ± 0.3	**0.8 ± 0.3**	<0.01
	ALA (C18:3n3)	0.3 ± 0.1	**0.2 ± 0.1**	<0.01
∑ PUFA (n6)		33.2 ± 3.2	**29.5 ± 3.8**	<0.01
∑ PUFA (n3)		4.1 ± 1.6	3.9 ± 1.4	NS
	O6/O3	9.2 ± 3.0	8.4 ± 2.7	NS
	AA/EPA	**20.9 ± 10.6**	39.4 ± 21.3	<0.01

Results are presented as mean ± SD. *p*-values < 0.05 were regarded as statistically significant. Shapiro–Wilk test and quantil-quantil-plots were used to test normal distribution of data. For comparisons between two groups, either a paired *t*-test or Mann–Whitney test was applied to assess statistical significance. Significantly lower values are marked in bold.

## 4 Discussion

Our observational study investigated the micronutrient status and fatty acid profiles of SARS-CoV-2 positive individuals, comparing them to a healthy cohort and stratifying within the infected group by hospitalization status to infer disease severity. These results contribute to a growing body of literature exploring the nutritional and metabolic dimensions of COVID-19 (12, 14, 18, 19, 25) and provide a solid foundation for further research.

We found that while overall micronutrient levels were comparable between SARS-CoV-2-positive and-negative groups, hospitalized patients showed significantly lower levels of vitamin D, selenium, zinc, magnesium, and iron than home care patients did. These reductions may reflect both preexisting nutritional insufficiencies and infection-induced redistribution or depletion, as previously described (2, 26, 27, 31). Although magnesium may increase in late-stage critical illness due to cell lysis or organ failure (28), this was not observed in our cohort. Instead, home care participants showed higher selenium and magnesium levels, possibly reflecting a lower inflammatory burden or early compensatory response. In contrast, lower magnesium levels in hospitalized patients may reflect increased metabolic demand and systemic inflammation, consistent with reports of declining magnesium levels with greater COVID-19 severity (28–30). Given the exploratory nature of our study, we have intentionally refrained from deeper pathophysiological interpretations.

Fatty acid profiles also showed distinct patterns. SARS-CoV-2 infection was associated with a marked decrease in saturated fatty acids and increases in polyunsaturated fatty acids, particularly omega-3 and omega-6 subclasses. These shifts are consistent with lipidomic studies indicating that viral replication and host immune modulation rely on fatty acid remodeling (14, 18, 19). Specifically, we observed a notable decrease in palmitic and stearic acids, along with an increase in omega-3 and omega-6 PUFAs in the SARS-CoV-2-positive cohort. These findings are consistent with those reported by Pérez-Torres et al. (14), who suggested that these fatty acids may be involved in viral membrane synthesis or host defense mechanisms. Pérez-Torres et al. (14) also noted elevated levels of oleic acid and AA, both of which are closely associated with inflammatory signaling, cytokine release, apoptosis, necrosis, and oxidative stress. Similarly, Sun et al. (19) found that higher levels of omega-3 and omega-6 PUFAs were linked to lower disease severity, a trend reflected in our study, where participants receiving home care therapy exhibited higher overall omega-3 and omega-6 levels than hosiptalized individuals. These alterations may therefore represent a complex, adaptive host metabolic response to viral pathogenesis (19).

Our study is subject to several limitations. Being cross-sectional in nature, it can only establish associations and not causality. The use of DBS, while practical, lacks hematocrit correction and may introduce variability in analyte concentration. The self-collection procedure, although guided, poses a risk of inconsistent sample quality. Additionally, the lack stratification by key variables (e.g., age, sex, comorbidities) may limit the granularity of our findings. The absence of urinary excretion data prevents comprehensive micronutrient balance assessment. Many of these constraints, however, must be viewed in light of the exceptional circumstances of the COVID-19 pandemic. Under these conditions, our approach represented the only feasible way to obtain robust and comparable biomarker data from a larger cohort.

Hospitalization was used as a proxy for disease severity, though no clinical scores (e.g., WURSS) or inflammatory markers were collected, limiting precision. Some participants also presented with comorbidities, further complicating interpretation. As a result, no conclusions about underlying biological mechanisms can be drawn. Although our results regarding alterations in fatty acid and micronutrient profiles remain broadly comparable with similar studies (14, 18, 19), which somewhat offsets this limitation. Additionally, confounding variables such as comorbidities, dietary intake, supplementation, and socioeconomic status were not fully controlled. Although the control group originated from a separate study sampling conditions were comparable, supporting a general level of comparability across groups. Data were analyzed using univariate statistics without adjustment for multiple comparisons, which may increase the risk of false positives.

Despite these limitations, our findings indicate that micronutrient status and lipid metabolism are altered in SARS-CoV-2 infection, with distinct patterns linked to disease severity. These results are consistent with prior studies and underscore the importance of integrated nutritional-metabolic assessment in infectious disease management (14, 18, 19). The present dataset, collected under the unique constraints of the COVID-19 pandemic, provides a valuable resource for future observational studies and meta-analyses on nutrition–infection interactions. While causality cannot be inferred, our results suggest that preventive consideration of adequate micronutrient levels may support immune readiness. These recommendations are made with caution, underscoring the need for controlled intervention studies and future research that accounts for confounders, integrates severity indices, and follows patients longitudinally from infection to recovery.

## 5 Conclusion

The findings of our observational study align with existing research, emphasizing the crucial role of adequate micronutrient levels, particularly magnesium, selenium, zinc, and vitamin D, in supporting immune function and potentially mitigating disease severity. These results underscore the dual nature of micronutrient deficiencies, which may both predispose individuals to infection and result from the increased physiological demands imposed by the immune response during illness.

In parallel, significant alterations in fatty acid metabolism were observed in the SARS-CoV-2-positive cohort, suggesting a complex interaction between viral replication and host lipid utilization. The observed changes may reflect both viral exploitation of lipid resources and host adaptive mechanisms to inflammation and recovery.

To our knowledge, this is the first study to jointly assess micronutrient and fatty acid profiles in SARS-CoV-2 patients across varying degrees of disease severity, directly compared with a healthy control group. These findings highlight the value of integrated metabolic profiling to advance the understanding COVID-19. We recommend the inclusion of those potentially immunoactive micronutrients in the surveillance of SARS-CoV-2 infections, of in further studies.

## Data Availability

The raw data supporting the conclusions of this article will be made available by the authors, without undue reservation.
